# Experts by Experience: Peer Support and its Use with the Homeless

**DOI:** 10.1007/s10597-017-0102-2

**Published:** 2017-02-07

**Authors:** Stephanie L. Barker, Nick Maguire

**Affiliations:** School of Psychology, University of Southampton, Building 44, University Rd, Southampton, SO17 1BJ UK

**Keywords:** Homelessness, Peer support, Quality of life, Drug/alcohol use

## Abstract

The homeless population has complex needs. Peers with experience of homelessness offer unique perspectives in supporting those experiencing homelessness. Peer support fostered and developed by professional organisations, termed intentional peer support (IPS), formalises this process. This review aims to assess the effectiveness of IPS as an intervention with young adults and adult homeless persons (including streetdwelling and those within services). PyscINFO, Web of Science, MEDLINE, and CINAHL were searched, resulting in ten studies, involving 1,829 participants. Peer support has significant impacts on quality of life, drug/alcohol use, and social support. Common elements of peer support are identified, suggesting possible processes that underlie effective peer support. Shared experiences, role modelling, and social support are suggested to be vital aspects of peer support and moderate changes in homeless clients. One study was deemed to have moderate/high quality; the remaining studies had low and moderate quality. Limitations of each are discussed.

## Introduction

Experts by experience represent individuals or groups who share a common experience of a social and health issues, such as homelessness. For clarity, those individuals or groups are classified as peers who have common experiences and can provide various types of support for someone who is ‘new’ to the experience or entering recovery (Dennis 2003; Salzer 2002). Alcoholics Anonymous (AA) characterises the most well-known of these peer groups that provide support for others, where more experienced members sponsor newcomers in the early stages of recovery. Indeed, this practice is also found in the mental health care systems; peers (also termed consumers or service users) have been providing mutual support since the 1800s (Faulkner et al. 2012). Peers are a positive source for fostering change and reconnecting the individual with the community (Repper and Carter 2011). The Substance Abuse and Mental Health Services Administration (SAMHSA) define peer support as “services [that] are delivered by individuals who have common life experiences with the people they are serving” and that they “have a unique capacity to help each other based on a shared affiliation and a deep understanding” of particular experiences (SAMHSA 2015, para 1).

Intentional peer support (IPS) is defined in a similar fashion, however, IPS is termed ‘intentional’ because it is fostered and developed by professional organisations. IPS can be either mentorship support or mutual support (Bradstreet 2006; Faulkner et al. 2012). Thus, studies that are using IPS may be using peers as client mentors or adjunct to services provided, such as combining peers and professionals in the delivery of services (e.g., Galanter et al. 1998). IPS models are quite diverse, organisations not only utilise peers in multiple ways, but peers may or may not be trained and/or paid for their work. Indeed, the common element is that peers share personal experiences with their clients and are viewed as distinct from professionals (Barker et al. 2017; Faulkner et al. 2012; Finlayson et al. 2016).

The prevalence of this type of intervention is displayed by their presence in numerous organisations and international guidelines, for example, in 2003, Wallcraft et al. identified over 716 programs that involve peers/consumers in England (Wallcraft et al. 2003). Further, researchers in Australia developed recommendations for the use of peer support within high-risk environments and Canadian advisory groups developed national guidelines of including those with lived experience in homelessness services (Creamer et al. 2012; National Lived Experience Advisory Council 2016).

Increasingly, IPS is used within homelessness services without a supporting evidence base. The Housing Act 1996 defines a homeless person as someone that lacks accommodation, cannot access accommodation, or resides in a vehicle or building which is unsuitable for occupation (Bennett et al. 2005, p. 9). This definition is useful in identifying those affected by homelessness but also results in a heterogeneous population, affecting families, youth, and single adults. However, young adults and adult homeless persons (including street dwelling and those engaged with services) are the focus of much of the research on this topic and this review.

Those who experience homelessness are qualitatively different than other populations. People who are homeless usually represent individuals that have the most complex, multi-morbid issues (Maguire et al. 2010). Compared to their domiciled counterparts, people living in temporary or emergency accommodations are eight times more likely to suffer from mental illness, while those who sleep rough are 11 times more likely to have a mental illness (Fitzpatrick et al. 2000).

IPS schemes are well-suited to supporting homelessness; peers have a unique ability to engage with those who are socially excluded (Pilote et al. 1996; Tulsky et al. 2000). Moreover, homeless people experience a “different world” of isolation and neglect, peers have experiential knowledge of this world which enables them to genuinely empathise and connect with the client (Barker et al. 2017, p. 13). Thus peers are a popular option for working with those currently experiencing homelessness. For example, Groundswell, a homeless charity in London, UK, that utilises peers to reduce the health disparity for those who experience homelessness, supports over 500 clients per year through their peer advocate scheme (Groundswell 2015). Peer supporters have a positive impact on clients experiencing homelessness by building relationships on “shared experience and [the] ability to empathise and develop a mutual trust and understanding” and provide various types of social support (Finlayson et al. 2016, p. 18).

Peers are currently aiding the homeless with health management, medication regimes, and acting as buffers for professionals (Deering et al. 2009; Fogarty et al. 2001; Gabrielian et al. 2013; Pilote et al. 1996; Rice et al. 2012; Tulsky et al. 2000). The literature examining the efficacy of this practice has been mostly supportive; various studies report that peers can help to reduce hospital admissions, relapses, increase coping skills, and improve overall quality of life for those with mental illness (Davidson et al. 2006; Salzer 2002; Solomon 2004; Wallcraft et al. 2003). However, a recent review suggests peers have only a minor positive impact on those with severe mental illness (Lloyd-Evans et al. 2014).

IPS with the homeless is currently being utilised internationally, but with limited literature to support it. Further, existing literature on IPS and homelessness has not been systematically reviewed. This review intends to begin filling in that gap by systematically exploring the efficacy of IPS with a sample of young adults and adults who are street dwelling and/or engaged with services.

## Objectives

Due to the lack of literature on homelessness and IPS, the initial aim was exploratory in nature: attempting to understand what the literature reveals about IPS and homelessness. Specifically, the review explored how IPS is currently being used with the homeless, the landscape of practice, outcomes of practice, and if IPS is a viable option for work with heterogeneous populations. The main research objective assesses the effectiveness of IPS with a homeless population. That is, peers *are* the intervention, not only delivering it. For the purpose of this review, homelessness is defined as single adults and/or young adults being without suitable accommodation including sleeping rough, in transient housing, hostels, sofa/couch surfing, living with friends, or other inappropriate accommodation.

The following objectives were explored during the search, as reflected in the search strategy terms:

Objective 1: How is IPS being used with those experiencing homelessness?

Objective 2: How effective is IPS with those experiencing homelessness?

## Methods

The review protocol was developed by the primary researcher and reviewed by two researchers. Studies that fulfilled one or more of these targets were considered:

1. Test the effectiveness of IPS with an adult and/or young adult homeless population.
2. Display common ingredients of IPS with a homeless population.
3. Evaluate IPS programs with a homeless population.

Studies that were not eligible had the following characteristics:

1. Testing the effectiveness of IPS with severe mental health, addictions, and/or health concerns in a non-homeless population.
2. Examining the cost effectiveness of peers in the workforce.
3. Examining outcomes without IPS.
4. Are not in English.

These criteria were selected because of the vast amount of research dedicated to IPS in sectors that prioritise issues that many homeless people face, but lack focus on treating homelessness. Literature in the search lacked focus on homelessness. The researchers included studies that had a primary intervention of IPS and its effects on those experiencing homelessness, with a minimum of 30% of the participants identifying as homeless. During the search process, it became clear that this arbitrary threshold needed to be added to the inclusion criteria, given that there is a breadth of evidence examining IPS within other contexts that, by chance, had participants experiencing homelessness in their sample. If we had chosen a 40% threshold, four studies that otherwise met criteria would have been excluded (e.g., Resnick and Rosenheck 2008; Tracy et al. 2012, 2014; van Vugt et al. 2012). A study considered for inclusion, as it met other criteria, had only 6% of its participants identifying as homeless and was excluded based on this threshold (Fukui et al. 2010). Thus, the intention of this cut-off is one of precision regarding the impact of IPS with a homeless population.

The search process refined the research question to examine the effectiveness of IPS with a homeless population. The search also revealed that IPS is currently being used with the homeless for various health interventions: TB, HIV, overdose prevention, and Hepatitis (Gabrielian et al. 2013; Tulsky et al. 2000; Wright et al. 2006).

The review searched MEDLINE, CINAHL, psycINFO, and Web of Science databases using keywords found in Table 1. Search terms were derived from keywords of relevant articles, consultation with a psychologist, and local homeless charities. Synonyms of peer-support included terms such as ‘consumer’ and ‘service user’ to accurately reflect terminology used in mental health and addiction services. This review attempted to account for publication bias by searching published, unpublished, and grey literature extensively (Song et al. 2010). The grey literature search was performed through Google Scholar, local and national charity publications/reports, and reports from conferences (full search strategy can be obtained by contacting first author).

**Table 1:** Search Terms

Operator	Definition
1. Keywords: Population	Adult OR over 18 OR older adult OR young adult
2. Keywords: Population	Homeless OR homelessness OR homeless person(s) OR rough sleeper OR rough NEAR/3 sleepers (specific to Web of Science)
3. Keywords: Intervention	Peer support OR peer OR service user OR consumer participation OR social support OR consumer OR peer counselling OR recovery
4. Keywords: Outcome	Effectiveness OR efficacy OR outcome OR impact OR treatment outcomes
5. Boolean Operator	1 AND 2 AND 3 AND 4
6. Language Limit	English
7. Selection	Removal of duplicates followed by PRISMA guidelines of article sifting: title sift, abstract sift, full-text sift, review reference lists and articles citing

PsychINFO via EBSCOHOST interface, 1944–2015; CINAHL Via EBSCOHOST interface, 1944–2015. Web of Science, 1950–2015; MEDLINE via OvidSP interface using all databases, 1946–2015 search conducted 02/10/15–02/28/15

The search was systematic, in two major stages, with the priorities of objectivity, transparency, and minimization of bias (Chambers et al. 2009). The first stage comprised of the researcher surveying titles and abstracts against the defined inclusion criteria to identify relevant studies to be reviewed in full. The second stage consisted of retrieving the full-text papers of the selected studies. The researcher documented study exclusions and reasons for exclusion at this stage. This process was also conducted in conjunction with another assessor, examining 10% of included studies and excluded ones, to ensure reliability.

## Results

**Fig. 1:**
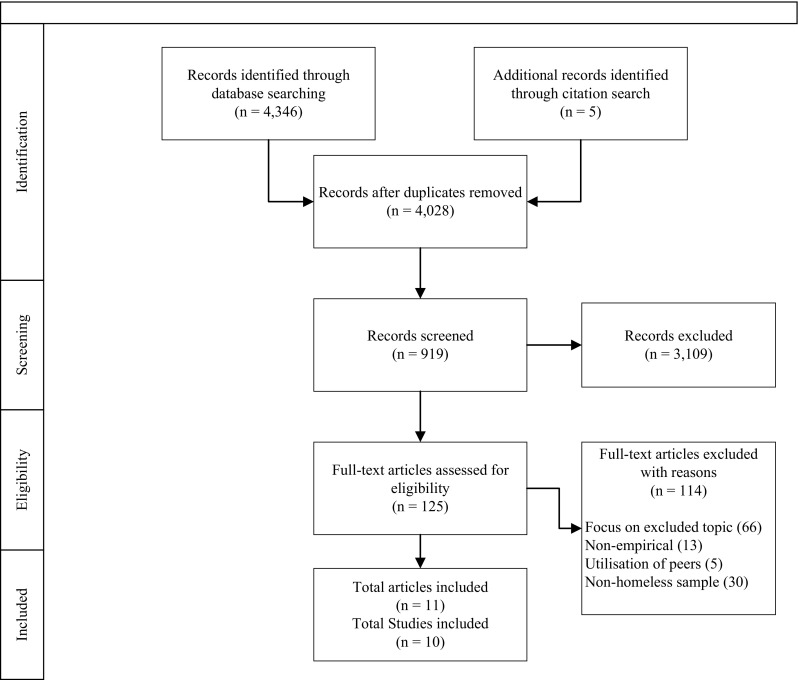
PRISMA flowchart: Screening of articles to be included

After the duplicates were removed, 4028 articles were identified for further review. Detailed information of this process is shown in Fig. 1, using the PRISMA flowchart (Moher et al. 2009). Eleven articles reporting on ten studies were included. Two articles reporting on the same data set were combined for the purposes of this review. Table 2 shows data extracted from the included studies that contain general information regarding the study participants and procedures. Data was also extracted specific to the research question including IPS definition, peer characteristics, how peers were used, and theories cited.

**Table 2:** Data extracted from included studies

Authors	Design	n	Methods	Tools	Interventions	Age	Sex	Race	Results	Peer support definition	How peer support is used	Peer traits
Bean et al. 2013 USA	Longitudinal	104	Surveys at baseline, 6 and 12 month	WHOQOL, arrest data	Housing first and peer support	56.06	72.2% Male	60% White 20% Black 15% N. American	Sig. change in QOL	NA	Part of housing intervention	Ex-homeless, mental illness, recovery
Boisvert et al. 2008 USA	Longitudinal	47	Interviews, pre/post (baseline and 9 months), and surveys	QOLR, MOS-SSS, VQ	Peer support community program	NA	NA	NA	Sig. change in relapse rates, mental health & functioning, perceived support/affiliation	“global change in lifestyle and identity that occurs in the social learning context …emphasizes beliefs and values essential to recovery”	To develop a socially responsible recovery community–everyone is expected to contribute	Role models who have sustained recovery
Felton et al. 1995 USA	Longitudinal	221	Baseline and 3 six month intervals	RSES, PSMS, BHS, CAARS, ICMES, ISEL, QOL, LPI, CSI	Peer supporters added to intensive case management vs case managers only and case managers + paraprofessionals	17% <30; 65% 30–50 18% 50+	60% Male	43% Black 42% White 15% Other	Peers equal to case-managers. Sig. outcomes in quality of life, social support, self-image, and community tenure	Make unique contributions that enhance service effectiveness, role modelling, provide empathy, sharing practical info. and coping strategies, and strengthening social supports	In conjunction with case-managers	Ex-consumers, with 8 weeks of training in counselling and self-help
Fors & Jarvis (1995) USA	Quasi-experimental; non-random	296	Survey at pre/post	Developed questionnaire	Peer led/adult led/ and non-intervention group	15	NA	NA	Peer-led groups were most effective, especially with younger sample	Mentors, prosocial aspect of life	Mentor, teacher	NA
Galanter et al. 1998 USA	Longitudinal	56	Urinalysis test for drugs of abuse 3 times over 4 months	Urinalysis tests	Peer and professional led group therapy	NA	60% Male	58% Black 41% White 32% Hispanic	69% achieved 3 clean urine tests	NA	Conjunction with professionals (peer led groups etc.)	NA
Resnick & Rosenheck 2008 USA	Quasi-experimental; non-random	321	Two cohorts: one treatment (n = 78) and one control (n = 218). Measured 3 times over 9 months	RAQ, MHCS, MDS, RAS, ADLS, GAF, ASI, BPRS, PTSD -Checklist-S, TLEQ, QOL, Participation	Vet-to-Vet; an addiction treatment delivered by peers compared to standard non-peer treatment	NA	95% Male	66% White	Treatment group improved on empowerment, confidence, functioning, and alcohol use	Benefit from interacting with people who have experiences similar life circumstances	In a program; delivering services	NA
Stewart et al. 2009 Canada	Cross-sectional	17	Within subjects	SPS, R-UCLA-LS, DS, PCI, HBS	4 Support groups/1:1 groups by peers and professionals	19	54% Male	60% N. American 27% White 13% Other	Sig. decreased loneliness. Qual. Results show increased support and coping	Peers as part of social support network by providing info, modelling, and encouragement	Part of group and 1:1	Ex-homeless youth
Tracey et al. 2012, 2014 USA	Cross-sectional	40	Clinical interviews, focus groups, training	SCID I, Designed fidelity measure	10 mentors with 30 mentees for 12 weeks	50.3	62% Male	40% Black 38% White 22% Hispanic	Alcohol/drug use decreased; No predictive factors of abstinence found (e.g. gender homelessness etc.)	Abstinence based relationship, role model, hope	Direct mentors	In recovery, 6 moths min. sobriety
Van Vugt et al. 2012 Netherlands	Longitudinal	10	With/without consumer providers, fidelity study, baseline and 9 months	Demographic, DSM-IV, HoNOS, CANSAS, WAS, DACTS	Consumer-providers impact on clients over time	41.6	71% Male	NA	Sig. mental state and functioning, un/met needs with personal recovery, and homeless days. Inverse relationship for hospital days (could be attuned to illness/needs)	Consumers as mental health professionals	Direct with clients/as their service workers	NA
Weissman et al. 2005 USA	Longitudinal	340	Peer support delivered 1 h/week with each participant baseline, 4, 8, and 12 months	Log books, ^a^Interviews	Peer supporters providing support on transitions, mentors, socialisation over 12 months	48	100%	75% Black 13% Hispanic 9% White 3% Other	Participants with peer mentors were more likely to follow-up in treatment and increased socialisation	“by virtue of their street smarts, engagement skills, peer support, positive role modelling, fighting stigma, and education of co-workers”	Peer mentors	Knowledge about recovery, prior group work experience, people skills + inclusion criteria

*WHOQOL* World Health Organization Quality of Life, *QOLR* Quality of Life Questionnaire, *MOS-SSS* Medical Outcome Study Social Support Survey—Focus on subscales: *EIS* Emotional/Informal support, *AS* Affectionate, *TS* Tangible, *VQ* Volitional Questionnaire, *RSES* Rosenberg’s Self-Esteem Scale, *PSMS* Pearlin and Schooler’s Mastery Scale, *BHS* Beck Hopelessness Scale, *CAARS* Client attitudes About Recovery Scale, *ICMES* Intensive Case Management Engagement Scale, *ISEL* Interpersonal Support Evaluation List, *QOL* Quality of Life Interview, *LPI* Life Problems Inventory, *CSI* Colorado Symptom Index, *RAQ* Recovery Outcome Questionnaire, *MHCS* Mental Health Confidence Scale, *MDS* Making Decisions Scale, *RAS* Recovery Assessment Scale, *ADLS* Activities of Daily Living Scale, *ASI* Addiction Severity Index, *BPRS* brief psychiatric rating scale, *TLEQ* Traumatic Life Events Questionnaire, *QOL* Lehman Quality of Life Scale, Participation in Vet-to-Vet. *SPS* Social Provisions Scale, *R-UCLA-LS* Revised. UCLA loneliness, Depression Scale, *DP* Depression Scale, *PCI* Proactive Coping Inventory, *HBS* Health Behaviour Survey, *SCID I* Structured clinical interview for DSM IV Axis I disorders, *HoNOS* Health of the National Outcome Scales, *CANSAS* Camberwell Assessment of Need Short Assessment Schedule, *WAS* Working Alliance Scale, *DACTS* Dartmouth Assertive Community Scale

^a^Interviews on employment status, housing, and substance use, overall QOL, social inclusion perception, social acceptance, symptoms of depression and anxiety

Nine studies were completed in the USA (Bean et al. 2013; Boisvert et al. 2008; Felton et al. 1995; Fors and Jarvis 1995; Galanter et al. 1998; Resnick and Rosenheck 2008; Tracy et al. 2012, 2014; Weissman et al. 2005). One was completed in Canada (Stewart et al. 2009) and the last study was from the Netherlands (van Vugt et al. 2012).

The review found that there was a wide variety of measures used by the ten studies. Most used standardised assessments and they were accurately reported in the articles. Two studies lacked in their reporting: Tracy et al. (2012, 2014) and Boisvert et al. (2008) did not provide references or vital information about their measures, so their outcomes are interpreted with caution. However, three studies used the same quality of life (QOL) measure and their outcomes can be directly compared against one another (Felton et al. 1995; Resnick and Rosenheck 2008; Weissman et al. 2005).

Included studies show baseline data for 1829 participants and complete data for 1341 participants; a loss of 488 or 27% of participants which is, overall, surprisingly low but this attrition affected some studies and confidence in their results drastically. None of the included studies examined adults experiencing homelessness exclusively; all incorporated some other identifying factor. The most common population drawn from was adults experiencing homelessness and dependent on substances: 494 participants from four studies (Boisvert et al. 2008; Felton et al. 1995; Galanter et al. 1998; Resnick and Rosenheck 2008; Tracy et al. 2012, 2014). The second most frequent population included adults who are homeless and diagnosed with mental health issues, with 425 participants. Homeless veterans comprised 313 participants, while 47 medically vulnerable homeless persons were the focus of another study. Lastly, there were 277 homeless youth/young people in this review. As some studies had complex sample populations, reflecting the complex needs of this population, participants may fall into one or more of the above categories, e.g., tri-morbidity—individuals who are homeless, substance dependent, and have mental health problems (Hewett and Halligan 2010).

All studies had peers as part of their intervention. Two studies had peers as mentors and assessed the impact on participants (Tracy et al. 2012, 2014; Weissman et al. 2005). Two studies included IPS as part of larger interventions. For example, in Bean et al. (2013) the combination of IPS, harm reduction, and housing first were assessed, while IPS was assessed in a community programme in another study (Boisvert et al. 2008).

Four studies compared peers to various groups. Felton et al. (1995) added peers to case manager teams, comparing outcomes from case managers only and case managers with paraprofessionals. Fors and Jarvis (1995) compared peers, adults, and a control group on the delivery of a harm reduction programme for homeless/runaway youth, Resnick and Rosenheck (2008) compared a peer-run, peer education programme to a control group, and, lastly, van Vugt et al. (2012) compared outpatient services with or without peers. The diversity of peer-programmes shows the complexity of this intervention, however, all programmes involved peers in a mentoring fashion, providing IPS to clients.

Two studies had peers working adjunctively with professionals/delivering services. These studies were still included as they were testing the effectiveness of peers within an IPS framework. One study tested a specialised clinic run by peers and professionals—treatment that is unusual—through urinalysis outcomes (Galanter et al. 1998) and the last one tested a peer-run and delivered peer education programme compared to treatment as usual—that is, treatment without peers (Resnick and Rosenheck 2008). If these studies were to be excluded, the review would have been lacking in results; they mirror how IPS is currently being used with a homeless population, reflecting a realistic climate of IPS and homelessness.

All studies had positive effects from IPS as an intervention; however, they vary on the size and confidence in those effects. Two studies found that outcomes with peers were comparable to the outcomes found with clinician-only groups (Felton et al. 1995; Resnick and Rosenheck 2008). Further, two studies had results that suggested peers were better than treatment as usual (Fors and Jarvis 1995; van Vugt et al. 2012).

The studies in the review show nine areas on which IPS has an impact: overall QOL, social support, physical and mental health, addiction/drug and alcohol use, life skills, homelessness, criminality, employment/finances, and attendance/interest. These areas are synthesised through the outcomes of each study. Each area found significant positive changes and/or nonsignificant changes relating to various outcomes, suggesting that further testing in this area is warranted. Significance values are reported here, when available. The general results of each area are reported below followed by an analysis of the quality of studies and the implications of each for the effectiveness of peers with homelessness.

### Overall Quality of Life

Four studies have results pertinent to this area (Boisvert et al. 2008; Felton et al. 1995; Resnick and Rosenheck 2008; Weissman et al. 2005). QOL was assessed through standardised measures for three studies and the fourth study used an unpublished measure. QOL in this category is defined as the overall satisfaction with life; being “mostly satisfied” or “pleased” with life. Significant results relate to a reduction in life problems (*p* < .05), increased satisfaction with living (*p* < .01), and a modest change to being mostly satisfied/pleased with life. Nonsignificant changes are also recorded for satisfaction with life overall from another study (*p* < .24), and on the unpublished measure used (Boisvert et al. 2008).

### Social Support

Social support is a common outcome measure; peers provided different types of support, increasing social relationships and social esteem. Four studies report a significant increase in aspects of social support, including increased belonging (*p* < .01), decreased loneliness (*p* < .05), increase in social relationships (*p* < .05) and general social support (Bean et al. 2013; Felton et al. 1995; Stewart et al. 2009; Tracy et al. 2012, 2014). Five types of social support, emotional; informational; tangible; appraisal; and companionship (Tardy 1985) are documented as being impacted positively by peer intervention. Specifically, three studies found that emotional and informational support increased: two with quantitative measures (*p* < .01 for both) and one through qualitative interviews (Boisvert et al. 2008; Fors and Jarvis 1995; Stewart et al. 2009). Three studies report an increase of tangible support through peer interventions (Boisvert et al. 2008; Felton et al. 1995; Fors and Jarvis 1995). Appraisal and companionship support (*p* < .05) had significant outcomes from two different study results (Boisvert et al. 2008; Felton et al. 1995). Lastly, social esteem significantly increased after the peer intervention for one of the studies (*p* < .01) (Felton et al. 1995).

This review also found that there were nonsignificant changes in the results related to social support. Three studies reported no changes regarding size/composition of social network, perceptions of social inclusion, social acceptance, and social relations after the peer intervention (Felton et al. 1995; Stewart et al. 2009; Weissman et al. 2005).

### Addiction/Drug and Alcohol Use

As is very common with a homeless population (Hewett and Halligan 2010), many of the participants reported dependence on drugs and/or alcohol. The samples had high rates of substance use and it was generally found that a peer intervention reduced harm related to addiction. Half of the included studies report positive outcomes in reducing drug and alcohol use (*p* < .05; *p* < .01), and reducing relapse rates (Bean et al. 2013; Boisvert et al. 2008; Galanter et al. 1998; Resnick and Rosenheck 2008; Tracy et al. 2012, 2014). Two studies found nonsignificant changes related to addiction; specifically, the amount of money spent on drugs and the amount of days using drugs or alcohol (Bean et al. 2013; Resnick and Rosenheck 2008).

### Physical and Mental Health

Physical health is shown to improve for participants across three studies. Participants reported an overall increase in health (*p* < .01) on quantitative measures from two studies (Bean et al. 2013; Felton et al. 1995). Qualitative interviews show that participants felt that they had increased their health promoting behaviours resulting from peer interventions (Stewart et al. 2009). The third study showed that hospitalisations increased during the intervention, which could be interpreted negatively, but researchers speculated that this increase was due to peers’ advocating and highlighting participants’ health needs (van Vugt et al. 2012). There are nonsignificant positive changes related to hospitalisations, A&E visits, inpatient admissions, and days spent in inpatient for two studies (Bean et al. 2013; Felton et al. 1995).

Concerning mental health, four studies saw an increase in overall functioning, psychological health (*p* < .05), and a reduction in psychiatric symptoms (*p* < .01) on quantitative outcomes (Bean et al. 2013; Resnick and Rosenheck 2008; Tracy et al. 2012, 2014; van Vugt et al. 2012). One study assessed mental illness symptoms through qualitative measures and participants report a reduction in depression and stress after the peer intervention (Stewart et al. 2009). Three studies found nonsignificant changes in recovery needs, PTSD and other psychiatric symptoms, and no change in perceived treatment of mental health (Bean et al. 2013; Resnick and Rosenheck 2008; van Vugt et al. 2012).

### Homelessness

Three studies report outcomes related to homelessness: decreases in the number of homeless days (*p* < .01), reduced relapse to homelessness, and reports of an overall improvement in environment (*p* < .01) (Bean et al. 2013; Boisvert et al. 2008; van Vugt et al. 2012). One study, however, did report that there was no significant change in homeless days and housing stability (Felton et al. 1995).

### Life Skills

Life skills developed as a concept for the outcomes from the studies referring to internal processes that contribute to recovery and were reported from half of the studies. For example, empowerment significantly increased (*p* < .05) from working with peers as mentors and educators (Resnick and Rosenheck 2008). Self-esteem improved from peer interventions on two studies—from qualitative and qualitative measures (Boisvert et al. 2008; Stewart et al. 2009). Peers also facilitate acceptance of illness and recovery, increasing efficacy, social skills, and coping as reported in three studies (Felton et al. 1995; Stewart et al. 2009; van Vugt et al. 2012). Lastly, there are nonsignificant changes related to recovery attitudes, empowerment over illness, and confidence as reported by one study (Resnick and Rosenheck 2008).

### Criminality

This area developed from two study results regarding arrests and contact with police; one study found a significant decrease in arrests (*p* < .01), another found non-significant changes in arrests and crime victimization (Bean et al. 2013; Felton et al. 1995).

### Employment/Finances

Employment is an outcome measure for three studies, with two significant results related to increased rates of employment and satisfaction with finances (*p* < .01; Felton et al. 1995; Weissman et al. 2005). The third study found a nonsignificant change in the number of days worked after the intervention (Resnick and Rosenheck 2008).

### Attendance/Interest

Four studies found that higher participation in treatment was a significant result of the peer intervention in each study. Tracy and colleagues (2012, 2014) found that higher rates of participation in the mentorship meetings/programme was significantly related to a reduction in drug and alcohol use (*p* < .01). One study found that their peer/professional intervention had high rates of attendance and another found that participants stayed in contact with professional services as a result of peer intervention (Felton et al. 1995; Galanter et al. 1998). Lastly, a qualitative outcome reports that participation and engagement were a central theme to the peer intervention (Boisvert et al. 2008).

## Discussion

### Study Quality

Utilising the Down and Black (1998) Quality assessment tool, scores are allocated to each individual study. The assessment tool is commonly used in measuring study quality in systematic reviews with non-random studies and is a recommended tool by the Cochrane Collaboration (Downs and Black 1998; Higgins and Green 2008). The tool has high internal consistency (Cronbach alpha > 0.69) for all subscales, save for the external validity scale, which has medium internal consistency (Cronbach alpha = 0.54). Ensuring reliability, this review utilised a second reviewer to score 10% of the included studies. Normally, each study is given a score out of 30, however, studies included in this review did not supply enough information for the question regarding power to be fully assessed, and thus each study was given a score of 1 or 0 if they had provided power information, resulting in a possible total score of 28.

The tool assesses studies for reporting quality, internal, and external validity. As the quality assessment was not used to exclude studies, the focus was on the score relating to validity questions. Sixteen questions evaluated bias associated with external and internal validity; these questions directed attention to the strength of study outcomes related to peer interventions with a homeless sample. Three items relate to external validity, which is the ability to generalise findings. Study bias is assessed by seven items, which examines bias in the intervention and outcome measure(s). Lastly, confounding and selection bias, which determines bias from sampling or group assignment, is measured by six items. In sum, each study was given a score out of 16 for their quality regarding generalisability, participant selection bias, and confounding variables (Higgins and Green 2008).

One study had the highest validity score, a score of ten or 63%, indicating that its results can be interpreted with the most confidence in this review (Felton et al. 1995). This study included a comparison of three treatment groups—case managers only; case managers and peers; case managers and paraprofessionals—on various outcomes for 104 participants over 2 years. This study is the most relevant in answering the research question by isolating peers and assessing their impact over a long period. The main significant outcomes assessed in this study include increased quality of life, social support, self-image and outlook, and community integration (Felton et al. 1995).

The next highest validity score is six or a 38% validity score. Five studies received this score suggesting that their outcomes must be interpreted with some caution. The remaining four studies scored lower than six: two of them scoring a 5, or 31%, and two scoring a 3 or 19% validity score. These lower scores indicate that their outcomes must be interpreted with extreme caution. A description of these scores and pertinent information is shown in Table 3.

**Table 3:** Downs & Black (1998) validity scores

	Downs and Black (1998) Validity Items Score	Effects size for Main Outcomes	Sample Size	Setting	Duration	Design
Felton et al. 1995	10	Large	104	Inpatient	24 Months	Longitudinal
Bean et al. 2013	6	None reported	47	Housing apartments	12 Months	Longitudinal
Fors and Jarvis 1995	6	Medium to large	221	Shelters	0.5 Months	Quasi-experimental
Resnick and Rosenheck 2008	6	Medium to large	296	VA premises	9 Months	Quasi-experimental
Stewart et al. 2009	6	None reported	56	Outpatient/drop-in	5.5 Months	Cross-sectional
van Vugt et al. 2012	6	None reported	321	Outpatient	9 Months	Cross-sectional
Tracey et al. 2012, 2014	5	Medium to large	40	Outpatient	6 Months	Longitudinal
Weissman et al. 2005	5	None reported	17	Outpatient	12 Months	Longitudinal
Boisvert et al. 2008	3	Medium to large	10	Inpatient	9 Months	Longitudinal
Galanter et al. 1998	3	Small to medium	340	Day treatment	4 Months	Longitudinal

### Common Elements of IPS

As there is minimal literature in this area, the authors used this review as an opportunity to build an understanding of common elements within IPS. IPS schemes for homelessness services are quite diverse; organisations utilise peers as formal, one-to-one mentors, informal supporters, group facilitators, and to link clients to professionals (Barker et al. 2017; Finlayson et al. 2016). Therefore, identifying common factors within this complex intervention will serve to develop the research programme and help focus future research in identifying if specific elements are critical to homelessness or mental health IPS interventions.

**Fig. 2:**
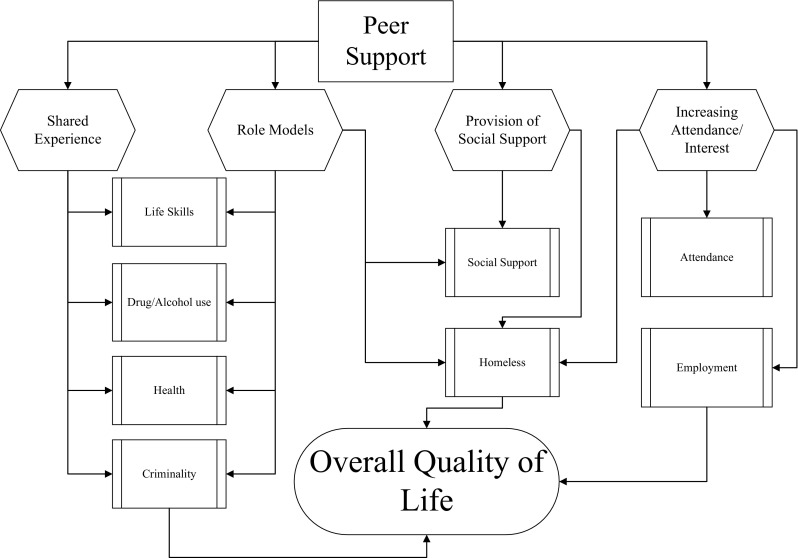
Common elements of IPS

Common factors reported from each included study are shown in Fig. 2. Elements were synthesized from the textual data of the included studies. We assessed why authors of the included studies chose IPS, their explanation of outcomes, and identified components. We approached the data qualitatively, searching for themes and patterns, then constructed ideas of the common elements of IPS. This is a tentative development and future work will attempt to develop ideas further. Common factors of IPS described in the studies include shared experiences, role modelling, social support, and attendance/interest.

Articles discussed how peers influenced overall quality of life through shared experiences of homelessness, mental illnesses, and addiction. Five articles cite how shared experiences serve to build trust and rapport, building prosocial relationships to facilitate recovery (Boisvert et al. 2008; Weissman et al. 2005; Felton et al. 1995; Resnick and Rosenheck 2008; van Vugt et al. 2012).

Another identified component of IPS includes role modelling; six studies discuss the role of social learning and social comparison (Boisvert et al. 2008; Felton et al. 1995; Fors and Jarvis 1995; Tracy et al. 2012, 2014; Weissman et al. 2005; Stewart et al. 2009). Mentors that possess similar traits are viewed as credible and provide a source of hope for clients to compare themselves, which enables motivation and self-efficacy for personal growth. Shared experience combined with role modelling is thought to improve life skills, reduce drug/alcohol use, increase health/healthy behaviours, and reduce criminality.

Three studies report that peers act as a source of social support and can impact participants’ feelings of belonging, normalising, and integration into a community, which enable the individual to develop life skills, increase their social network, and reduce homeless days (Boisvert et al. 2008; Felton et al. 1995; Stewart et al. 2009). Lastly, the concept of attendance/interest was developed as an aspect of IPS from four studies as their attendance rates and participants’ interest in the peer interventions remained high (Galanter et al. 1998; Tracy et al. 2012, 2014; van Vugt et al. 2012; Stewart et al. 2009). Although attrition was an issue, authors mentioned that the control groups suffered more, and reported that, similar to previous literature, peers fostered interest in the intervention, facilitating retention (Pilote et al. 1996; Tulsky et al. 2000). Higher attendance and interest for treatment is hypothesised to be linked to a reduction in the number of homeless days and increased employment. These findings support previous research examining common elements of peer support; Salzer (2002) and Campbell (2008) also assert that shared experiences, role modelling, and social support are integral to peer support.

Obviously, IPS is a complex process and the diverse outcomes reported suggest that it can positively affect various aspects of an individual’s life. However, the available evidence proposes that there are significant change mechanisms involved. The UK Medical Research Council states that only through careful understanding of the causal mechanisms involved in a complex intervention can it be applied to different settings and its effectiveness understood (Craig et al. 2008; Moore et al. 2015). One potential change mechanism involves the strength of the relationship between the client and peer supporter; just as the therapeutic relationship is vital to meaningful change in psychotherapies, the peer-client relationship is influential to developing behavioural and cognitive changes. This dynamic relationship provides multiple types of support and role models that positively impact recovery from multiple issues. It is argued that homelessness experience is integral to effective IPS in homeless services. Rough sleeping and homelessness is a unique experience which involves exclusion from every aspect of society, that a shared experience of addiction or mental illness would not suffice in building the relationship between peers and clients in a homeless setting (Barker et al. 2017). However, more research into IPS and homelessness is required to assess if IPS interventions for homeless populations are qualitatively different than those with mental illness or addictions.

### Limitations

#### Limitations of Included Studies

Most of the included studies had methodological issues; hence, the lower scores on the Downs and Black (1998) assessment tool (see Table 3 for detailed validity scores). For example, the most common limitation cited was the lack of randomisation. Only one of the studies was able to randomise participants to their interventions. Study authors discussed the impossibility of randomisation in the context of their study—participants were already assigned to certain staff or the study lacked comparison groups. One study that did randomise its participants recruited after they had completed a specific treatment and were piloting an IPS intervention (Weissman et al. 2005). None of the studies blinded participants and only one (Felton et al. 1995) blinded those measuring the outcome measures, but the rest did not avoid this potential scoring bias. In addition, none of the studies reported power. The individual studies reported their limitations on sample size, lacking control groups, attrition, and non-randomisation.

Limitations identified by the researcher are described as follows: Boisvert et al. (2008) used a QOL measure that was not cited in their references. Upon further investigation, it was found that this tool, the Quality of Life Rating Scale, is an unpublished measure and information about its validity and reliability were reported at a conference. This tool might be adequate but there was not enough information provided to have confidence in its outcomes.

Fors and Jarvis (1995) compared peers to adults in the delivery of a drug harm-reduction program; however, their comparison groups were extremely unbalanced. Indeed, the peer group had 173 participants while non-peers had only 34, and the control had 14. It is not surprising that the only group with significant results are peers. Unfortunately, the authors did not explain why there was such discrepancy between sample sizes. Lastly, Weissman et al. (2005) suffered such extreme attrition from their control group that they completely excluded that data from the report. The results of this study were lacking as there was very little reported.

It is possible that addressing these issues may show that the nonsignificant changes develop into significant ones; however, further work is needed to confirm that assertion. At a minimum, further testing would give greater confidence in the results. The presence of all these limitations speaks to the complexity of completing research with this population, which is also represented by the lack of literature in this area. Despite these issues, all included studies had significant outcomes from their peer intervention on a homeless sample. This provides evidence that IPS can have an impact, but work must be conducted to support these results.

#### Limitations of Review Methodology

This review was limited by the threshold of including articles that had samples with at least 30% of them identified as homeless. While this criterion was used to ensure that articles that had a meaningful focus on homeless populations, it is an arbitrary proportion and limited the included studies in a way that may have been biased (e.g., focusing on variables already under scrutiny in the homelessness field, such as mental illness or addiction). To the best of the authors’ knowledge, such a threshold has not previously been used and could have weakened the results in exploring IPS interventions with homeless populations; however, the threshold was required for the focus of this review. Further, the narrative synthesis of identifying common elements involved a level of abstraction. Therefore, the textual data could have been interpreted differently by another researcher. However, these common elements were firstly identified by frequency and then discussed and agreed upon with the contribution of two reviewers.

Although effort was made to include a second reviewer when possible, the search was completed by one researcher. The second reviewer was provided with articles selected for inclusion and exclusion to assess the inclusion criteria and to help focus the review. Further, the second reviewer was involved in assessing the quality of included studies. This also provided an opportunity for the researcher to assess any ambiguous articles for inclusion. Having only one researcher complete the search could have biased the included studies, however the wide scope of the search helped to reduce this possibility. Lastly, this review was limited by available resources to include articles written in English, the inclusion of other languages could have strengthened the findings and resulted in a more global perspective of IPS with homeless populations.

## Conclusions

This review found 11 articles describing ten studies that examined the effectiveness of IPS with a homeless population; demonstrating limited evidence of IPS with a homeless population. Positive outcomes relate to the improvement of the participants’ overall QOL, specifically, the reduction of drug/alcohol use, improved mental/physical health, and increased social support. Results were grouped into eight outcomes related to QOL, and each of these areas had conflicting results. Evidence in this area is underdeveloped and this was the first review to examine IPS with a homeless sample. The embryonic nature of this topic inherently suggests that more evidence is required. This review attempted to begin that process and inspire more research in this area, especially since services are currently using IPS in treatment for the homeless. Common elements of IPS were identified from the included studies suggesting that IPS works through components of shared experience, role modelling, providing social support, and increasing attendance/interest. Those four components are thought to moderate overall QOL through the eight outcomes reported. These findings signify the value of creating prosocial and intentional relationships between clients and peers, and acknowledge the complexity and challenges of applying the appropriate IPS processes thus resulting in varying levels of successful outcomes.

The results show that IPS can have a positive impact on outcomes for homeless people. Based on the evidence in this review, homeless organisations utilising peers should focus their outcomes on the areas where peers are shown to have impact, such as reduction of drug/alcohol abuse/use, increasing mental and physical health, and increasing social support. Practical applications of these results pertain to the training of peers whereby training sessions focus on the common elements and the identified outcomes. For example, peers could learn how to use their shared experiences in a manner that models recovery from homelessness. Further, peers may learn about the different types of social support and how to provide each type. Other sessions could increase peers’ knowledge about drug/alcohol use, mental and physical health, and how important social support is to recovery from homelessness. Policy may be informed by this research towards implementing IPS into practice regulations, however with caution, much more research is needed to ascertain clearly defined peer interventions and their impact on homeless populations. Future research could address the identified limitations and examine the effectiveness of peers with the homeless through robust experimental measures. Future research in this area will help provide evidence for a practice that already is in use and further our understanding of IPS and its complex processes.
